# Solidaritätsorientierungen und soziale Positionen. Klassenhabituelle Haltungen zu Sozialstaat und Geflüchteten in Österreich

**DOI:** 10.1007/s11609-022-00473-x

**Published:** 2022-06-14

**Authors:** Carina Altreiter, Jörg Flecker, Ulrike Papouschek

**Affiliations:** 1 Institut für Soziologie und empirische Sozialforschung, Wirtschaftsuniversität Wien, Welthandelsplatz 1, 1020 Wien, Österreich; 2 Institut für Soziologie, Universität Wien, Rooseveltplatz 2, 1090 Wien, Österreich; 3 Wien, Forschungs- und Beratungsstelle Arbeit, Asperbrückengasse 4, 1020 Wien, Österreich

**Keywords:** Solidarität, Habitus, Spaltungslinien, Soziale Klasse, Sozialstaat, Solidarity, Habitus, Lines of cleavage, Social class, Welfare state, Solidarité, Habitus, Lignes de partage, Classe sociale, Etat-providence

## Abstract

Auf Basis qualitativer Daten untersucht der Beitrag, welche Bedeutung der Klassenlage für die in der österreichischen Gesellschaft vorzufindenden Solidaritätsorientierungen zukommt, v.a. im Hinblick auf sozialstaatliche Arrangements und die Solidarität mit Geflüchteten. Das empirische Material wurde in einer zwischen 2016 und 2019 durchgeführten Studie in Österreich erhoben und umfasst insgesamt 48 Interviews. Die Aussagen werden zunächst mithilfe einer Heuristik von fünf Solidaritätsdimensionen zu einer Typologie von sieben Solidaritätskonfigurationen systematisiert und daraufhin im Hinblick auf die Relevanz der jeweiligen Klassenlage der Befragten differenziert. Mit Rekurs auf Bourdieu wird dabei nicht nur auf die objektive Klassenlage Bezug genommen, sondern auch auf die latenten Wirkungsmechanismen der Klassenherkunft im Sinne eines Klassenhabitus. Auf diese Weise kann sowohl bei nicht-privilegierten als auch bei privilegierten Befragten gezeigt werden, wie die jeweiligen Solidaritätskonfigurationen von der Klassenzugehörigkeit beeinflusst, wenn auch nicht determiniert sind. Im Ergebnis zeigen sich aufschlussreiche Unterschiede v.a. in Hinsicht auf Reichweite, Bedingungen und Gerechtigkeitsprinzipien der jeweiligen Solidaritätsorientierung. Es wird deutlich, dass der Fokus auf Klasse das Verständnis von Ein- und Ausschlüssen im Hinblick auf die sozialstaatliche Solidargemeinschaft plausibilisiert.

## Abstract (en)

The article examines the importance of social class in shaping solidarity orientations in Austria, especially with regard to welfare state arrangements and solidarity with refugees. It builds on 48 interviews conducted between 2016 and 2019. Distinguishing multiple dimensions of solidarity, the paper builds a typology of seven solidarity configurations, which are then systematically related to the class position of interviewees articulating them. In that context, not only objective class positions, but also latent mechanisms of habitus formation are taken into account. In so doing, the paper indicates how solidarity configurations of both privileged and non-privileged interviewees are shaped, but not determined by class. This casts new light on meaningful differences in the scope, conditions and underlying normative principles of distinct solidarity configurations. Class is key to understanding how attitudes towards the community of welfare solidarity are tied to specific preferences as to who should be included in and excluded from that community.

## Résumé

Sur la base de données qualitatives, l’article examine l’importance de la situation de classe pour les orientations solidaires dans la société autrichienne, notamment en ce qui concerne les dispositions de l’État-providence et la solidarité avec les réfugiés. Le matériel empirique a été recueilli dans une étude menée en Autriche entre 2016 et 2019 et comprend un total de 48 entretiens. Les énoncés sont d’abord systématisés à l’aide d’une heuristique de cinq dimensions de solidarité en une typologie de sept configurations de solidarité, puis différenciés selon la pertinence de la situation de classe respective des répondants. En recourant à Bourdieu, il est fait référence non seulement à la situation de classe objective, mais aussi aux mécanismes d’action latents d’origine de classe au sens d’un habitus de classe. On peut ainsi montrer comment les configurations de solidarité respectives des enquêtés non privilégiés et privilégiés sont influencées, bien que non déterminées, par l’appartenance à des classes sociales. Le résultat montre des différences révélatrices notamment en ce qui concerne la portée, les conditions et les principes de justice de l’orientation solidaire respective. Il devient clair que l’accent mis sur la classe rend plausible la compréhension des inclusions et des exclusions à l’égard de la communauté de solidarité sociale et étatique.

## Einleitung

Solidarität galt und gilt als ein zentraler Grundpfeiler der europäischen Arbeiter*innenbewegung. Seit ihrer Entstehung im 19. Jahrhundert stand der Begriff für die Verbundenheit der arbeitenden Klassen und den gemeinsamen Kampf für eine gerechtere Welt, ermöglicht durch die Einsicht, dass man eine „objektive“ Klassenlage teilte. Eine der zentralen, bis in die Gegenwart fortwirkenden Errungenschaften dieser Kämpfe sind wohlfahrtsstaatliche Arrangements, die heute sämtliche Staaten in Europa kennzeichnen, auch wenn sie sich hinsichtlich des Ausmaßes ihrer Ausgestaltung und Ausdehnung zum Teil stark unterscheiden. Das europäische Solidaritätsarrangement fordistischer Prägung – mit seiner einigermaßen stabilen Kombination von (National‑)Staatlichkeit, (Vollzeit‑)Erwerbsarbeit und sozialer Absicherung – geriet jedoch bekanntlich mit der Rückkehr ökonomischer Krisen im auslaufenden 20. Jahrhundert und der in deren Zuge seit den 1980er-Jahren hegemonialen neoliberalen Agenda in Bedrängnis. Es folgten staatliche Kürzungsprogramme und Deregulierungen des sozialen Sektors durch Privatisierung und Liberalisierung in so gut wie allen europäischen Staaten. Bis heute dominiert diese Logik vielfach politische Debatten und Entscheidungen, auch wenn der Ab- bzw. Umbau des Sozialstaates keineswegs überall gleich und linear verläuft. So konnten beispielweise in Deutschland oder Österreich als Reaktion auf die Finanz- und Wirtschaftskrise – oder auch jüngst im Kontext der COVID-19-Pandemie – Ausweitungen der Maßnahmen zur sozialen Absicherung (z.B. Einführung von Kurzarbeitergeld) beobachtet werden.

Als „institutionalisiertes Solidaritätsarrangement“ (Lessenich 1999, S. 25) entstand der Sozialstaat als absichernde Infrastruktur einer jungen kapitalistischen Gesellschaftsordnung, mit der für die arbeitende Bevölkerung manifeste Existenzgefährdungen einhergingen, etwa infolge des Ausfalls der eigenen Arbeitskraft durch Krankheit, Alter oder Arbeitslosigkeit.^1^ Seine Existenz verdankt er historisch gesellschaftlichen Kämpfen, die die kapitalistische Gesellschaftsordnung seit ihrer Entstehung begleiteten. Insofern verwundert es nicht, dass dieses nationalstaatlich organisierte Solidaritätsarrangement auch heute noch Gegenstand von Aushandlungen und Verteilungskämpfen ist. Auch wenn die Hauptkonfliktlinie in der Auseinandersetzung um Ausbau, Abbau und Ausgestaltung der Sozialpolitik zwischen Kapital und Arbeit verläuft, geht es dabei vielfach auch um die Grenze zwischen Innen und Außen im Sinne der Zugehörigkeit zur sozialstaatlichen Solidargemeinschaft. Gegenstand der Auseinandersetzung sind nicht nur materielle Ansprüche, sondern auch die Voraussetzungen, die für die Aufnahme in die bzw. für den Verbleib in der Solidargemeinschaft zu erfüllen sind. Es geht mithin auch um Problematiken der Anerkennung und um Deutungshoheiten hinsichtlich der Frage, wer überhaupt das Recht hat, legitime Ansprüche vorzubringen. Und relevant sind in diesem Zusammenhang nicht nur Programme und Botschaften von Parteien und Interessenvertretungen, sondern – in Wechselwirkung mit diesen – ebenso die politischen Orientierungen der Bürger*innen.

Der folgende Beitrag analysiert, welche Bedeutung Klassenlagen in der Konfiguration von sozialstaatlichen Solidaritätsorientierungen zukommt. Es wird nach dem Zusammenhang zwischen sozialer Position und den wahrgenommenen normativen Verpflichtungen zur Unterstützung anderer bzw. der Befürwortung sozialstaatlicher Absicherung gefragt. Grundlage dafür bildet die Studie „Solidarität in Zeiten der Krise“, die zwischen 2016 und 2019 in Österreich und Ungarn mit dem Ziel durchgeführt wurde, den Wandel von politischen Orientierungen und insbesondere Solidaritätsvorstellungen vor dem Hintergrund der „Flüchtlingskrise“ von 2015 zu analysieren (Altreiter et al. 2019).^2^ Deren Folgewirkungen dienten der von 2017 bis 2019 im Amt befindlichen rechts-konservativen Koalitionsregierung von Volkspartei und FPÖ als Legitimation für einen Umbau sozialstaatlicher Arrangements.^3^ Unter dem Slogan „Die Zuwanderung in unser Sozialsystem stoppen“ (Kronen Zeitung 2017) versuchte die Regierung Kurz I, Einschränkungen des Sozialstaates als Maßnahmen zu seiner Verteidigung erscheinen zu lassen, indem sie wohlfahrtsstaatliche Leistungen österreichischen Staatsbürger*innen vorbehielt. Dazu propagierte sie ein eng ausgelegtes Leistungsprinzip („Wer arbeitet, darf nicht der Dumme sein“) und heizte die Debatte um den Missbrauch von Sozialleistungen an, wobei sie die klassisch neoliberale Behauptung anführte, deren Finanzierungsmöglichkeiten seien infolge von „Sozialmissbrauch“ an Grenzen gestoßen (Flecker et al. 2018, S. 248). Realiter wurde die vormalige „Bedarfsorientierte Mindestsicherung“ durch die „Sozialhilfe Neu“ ersetzt, die Kürzungen für Mehrkinder-Familien vorsah sowie auf die explizite Benachteiligung von Migrant*innen und Menschen mit begrenzten Deutschkenntnissen abzielte. Angedacht war darüber hinaus eine Kürzung der Bezugsdauer der Arbeitslosenversicherung, insofern die prinzipiell nicht befristete einkommensabhängige Notstandshilfe in die Grundsicherung integriert werden sollte.

Vor diesem politischen Hintergrund galt das Interesse der Studie den in der österreichischen und ungarischen Bevölkerung vorherrschenden Perspektiven auf den Sozialstaat. Die nachstehende Analyse rekurriert auf 48 qualitative Interviews mit erwerbstätigen und erwerbslosen Personen aus dem österreichischen Pool der Studie und legt den Fokus auf die Frage, wo und wie „Klasse“ in den Interviewaussagen zu sozialstaatlicher Solidarität mit anderen Identifikations- und Distinktionsmechanismen wie Ethnizität und Nationalität zusammenwirkt. Es geht dementsprechend um die Frage, welche manifeste oder latente Rolle die Klassenlage in den variierenden Verständnissen von Solidarität spielt, die die Befragten in Bezug auf den Sozialstaat zu erkennen geben. Dabei konzentrieren wir uns insbesondere auf Aussagen zur sozialstaatlichen Absicherung von Erwerbsarbeitslosen und Geflüchteten. Damit wollen wir zu einer Forschungsdebatte beitragen, die nach der sozialen Genese solidarischer Orientierungen fragt, auch mit Blick auf politische Orientierungen (vgl. u.a. Hochschild 2017; Vester 2017; Dowling et al. 2017). Mit der Integration einer Klassenperspektive zielen wir in diesem Zusammenhang auf die Frage, wie sich „der Problemrohstoff, der aus sozialen Klassenpositionen hervorgeht“ (Dörre 2020, S. 39), – wie zum Beispiel Abwertungserfahrungen oder Abstiegsbedrohungen – in solidarische Orientierungen übersetzt. Wir lenken die Aufmerksamkeit mithin auf soziale Positionen, damit assoziierte Asymmetrien, Macht- und Herrschaftsbeziehungen sowie schließlich auf die damit verbundenen latenten Auseinandersetzungen um Solidaritätsorientierungen.

Nach einer Darstellung der methodischen Anlage der empirischen Untersuchung (2) stecken wir das theoretische Feld der Analyse ab (3), wobei es zunächst um die Auseinandersetzung um den Solidaritätsbegriff und seine sozialwissenschaftliche Anwendbarkeit geht (3.1). In der Folge wird das dem Beitrag zugrundeliegende Verständnis von Klassenlagen skizziert (3.2) und die für die Untersuchung leitende Typologie der Solidaritätsorientierungen vorgestellt (3.3). In der analytischen Konzeption von Klasse beziehen wir uns auf Pierre Bourdieu und berücksichtigen entsprechend nicht nur die objektive Lage bzw. ein gegebenenfalls manifestes Klassenbewusstsein, sondern auch die latenten Wirkungsmechanismen der Klassenherkunft im Sinne eines Habitus. Im empirischen Hauptteil (4) präsentieren wir darauf aufbauend detailliert die den jeweiligen Typen eigenen Solidaritätskonfigurationen und die darin enthaltenen Ein- und Ausschlüsse im Hinblick auf die sozialstaatliche Solidargemeinschaft (4.1–4.7). Es wird insbesondere deutlich gemacht, wie Klasse als analytisches Konzept dabei helfen kann, die mit den Solidaritätskonfigurationen assoziierten Deutungsmuster in ihrer sozialen Bedingtheit zu interpretieren. Wenn sie auch nicht die alleinige Erklärungsgrundlage bilden kann, so macht die Analyse deutlich, dass der Klassenzugehörigkeit erkennbar eine zentrale Bedeutung zur Erklärung der jeweiligen Solidaritätsorientierungen der Befragten zukommt (5).

## Methodische Anlage der Studie

Der Beitrag basiert, wie eingangs erwähnt, auf einer zwischen 2016 und 2019 in Österreich und Ungarn durchgeführten empirischen Studie, welche die Auswirkungen des in Zeiten der Krise beschleunigten sozioökonomischen Wandels auf politische Orientierungen zum Gegenstand hatte. Parallel zu Erhebungen in Ungarn haben wir auf der Basis der Triangulation einer quantitativen Erhebung (*N* = 1250) und von qualitativen Interviews (*N* = 48) subjektive Verarbeitungsformen von Krisenerfahrungen und ihre Überführung in unterschiedliche Solidaritätskonfigurationen in Österreich untersucht. Die Einbeziehung von Klassenlagen entwickelte sich im Zuge der qualitativen Erhebung – unter anderem angesichts von Leerstellen, die sich bei der Interpretation der vorgelagerten quantitativen Befragung herausstellten. Diese Befragung enthielt zwar grundlegende sozial-strukturelle Indikatoren, die wir jedoch als nicht ausreichend für eine Klassenanalyse im engeren Sinne einschätzten und aus diesem Grund nicht in die folgende Analyse einbezogen haben.

Der vorliegende Beitrag rekurriert auf den österreichischen Part der qualitativen Erhebung der Studie, für die zwischen Februar und August 2018 insgesamt 48 Interviews mit Personen im erwerbsfähigen Altern in ganz Österreich durchgeführt wurden. Bei der Auswahl der Interviewpartner*innen waren wir bemüht, eine möglichst große Bandbreite sowohl von sozio-demographischen Merkmalen – wie Bildung, Beruf, Alter, Geschlecht oder Wohnort – als auch politischen Orientierungen abzudecken. Dabei dienten uns die Ergebnisse der quantitativen Erhebung, aber auch Analysen einer Vorgängerstudie (Flecker 2007) als Orientierung für die Auswahl der Fälle und die Zusammenstellung des Samples. Es ging nicht darum, statistische Repräsentativität zu erlangen, die eine qualitative Forschung per se weder einlösen kann noch will, sondern darum, das Wissen über die Bedeutung bestimmter Merkmale aus den bisherigen Befunden – wie z. B. unterschiedlicher beruflicher Erfahrungen, der regionalen Herkunft oder des Bildungshintergrunds – gezielt für die Erhebung zu nutzen (Przyborski und Wohlrab-Sahr 2014, S. 182 f.). Darüber hinaus war es das Ziel, Fälle mit sowohl minimalen als auch maximalen Unterschieden hinsichtlich der Erfahrungen, aber auch der Solidaritätsorientierungen in die Studie zu integrieren, um daraufhin deren Varianzen auszuloten. Ähnlich dem Vorgehen des „theoretischen Sampling“ in der Grounded Theory (Strauss und Corbin 1996, S. 148 ff.; Glaser und Strauss 1968) wechselten wir in einem zyklischen Vorgehen Phasen der Datenerhebung mit Phasen der Auswertung, um daraus wiederum Schlüsse für die Auswahl der nächsten Fälle treffen zu können.

Die Gespräche folgten den Prinzipien des problemzentrierten Interviews (Witzel 2000). Diese Form des leitfadengestützten Interviews eignet sich insbesondere für qualitative Forschungen, in denen es eine thematische Fokussierung gibt. Der Leitfaden steckte relevante Aspekte ab, ließ den Interviewpartner*innen aber größtmögliche Freiheiten in ihren Narrationen. Die Gespräche dauerten zwischen 60 und 180 Minuten und umfassten Bildungs- und Berufslaufbahn, aktuelle Lebens- und Wohnsituation, ehrenamtliches Engagement, Fragen zu gesellschaftlicher Anerkennung, Einschätzungen zum Sozialstaat und zum sozialen Zusammenhalt sowie Eindrücke im Zusammenhang mit den Fluchtbewegungen von 2015. Für jedes Interview wurde darüber hinaus ein Kurzfragebogen zu sozialstatistischen Daten ausgefüllt.

Insgesamt wurden 21 Frauen und 27 Männer befragt. 18 Befragte verfügten über einen Hochschulabschluss, 14 hatten Matura (Abitur), 15 eine Lehre oder eine berufsbildende mittlere Schule abgeschossen, eine Person verfügte über einen Pflichtschlussabschluss. 33 Befragte waren unselbstständig beschäftigt, neun Personen selbstständig, und sechs Personen zum Zeitpunkt des Interviews erwerbslos oder in einem Beschäftigungsprogramm des Arbeitsmarktservice (siehe Tab. 1).

**Table 1:** 

*Altersverteilung*	
20 bis 29	9
30 bis 39	8
40 bis 49	20
50 bis 59	8
60 bis 65	3
Summe	48
*Branchen*	
Landwirtschaft	1
Herstellung von Waren	8
KFZ-Reparaturen	1
Beherbergung und Gastronomie	4
Information und Kommunikation	6
Öffentliche Verwaltung	5
Erziehung und Unterricht	3
Gesundheit und Sozialweisen	7
Freiberufliche wissenschaftliche/technische Tätigkeiten	6
Finanzdienstleitung	1
Erwerbslose	6
Summe	48

Quelle: Eigene Darstellung

Die Gespräche wurden mit Einverständnis der Interviewten aufgezeichnet und vollständig transkribiert. Im Zuge dessen wurden auch sensible personenbezogene Daten anonymisiert. Die Analyse der Interviews folgte einem mehrstufigen Verfahren. Dafür orientierten wir uns an Vorschlägen von Flick (1995) sowie Mühlfeld et al. (1981), die für die Analyse einer größeren Anzahl von Fällen und auch für die Bildung von Typen geeignet schienen. Dazu wurden zunächst zu allen Interviews ausführliche Einzelfallauswertungen erstellt. In diesem Analyseschritt wurde rekonstruiert, in welchen Aussagen Solidarität explizit oder implizit zum Thema gemacht wurde und welche Deutungsmuster damit verbunden waren. Mithilfe der Techniken der rekonstruktiven Sozialforschung, darunter das hermeneutische Verfahren der Feinstrukturanalyse (Froschauer und Lueger 2003), wurden die spezifischen Solidaritätskonfigurationen jedes Falles herausgearbeitet. Die rekonstruierten Aussage- und Deutungsmuster dienten als Grundlage für kontrastierende Fallvergleiche – dem zweiten Analyseschritt. Auf der Grundlage dieser Fallvergleiche wurde anschließend eine Typologie entwickelt.

## Theoretischer Rahmen

### Solidarität als analytisches Konzept

Solidarität bezeichnet eine besondere Form der Bindung zwischen Menschen, die auf Gemeinsamkeiten – wie geteilte soziale Lage oder Werte – beruht, aus denen die Beteiligten eine moralische (Selbst‑)Verpflichtung zum gegenseitigen Beistand ableiten. Der Begriff lässt sich historisch auf die römische Antike zurückführen, als „obligatio in solidum“ die unbeschränkte Verpflichtung jedes Familienmitglieds bedeutete, die Schulden der Familie bzw. anderer Familienmitglieder zurückzuzahlen – oder anders ausgedrückt: die Haftung der Gemeinschaft und der ihr Zugehörigen für die Schulden eines jeden anderen Mitglieds (Bayertz 1998, S. 11). Wie der Begriff der „obligatio“ andeutet, handelte es sich ursprünglich um ein Rechtskonzept: Das finanzielle Einstehen der Familienmitglieder füreinander war einklagbar und galt unabhängig von individuellen Präferenzen und Sympathien. Erst infolge der Entwicklung des säkular-bürgerlichen Nationalstaats der europäischen Moderne und der Idee horizontaler „Brüderlichkeit“ löste sich das Verständnis von dieser ursprünglichen Assoziation mit einer Rechtsverpflichtung. Als *moralische* Selbstverpflichtung freier Individuen zum wechselseitigen Einstehen füreinander gilt Solidarität seit dem 19. Jahrhundert als freiwillig. Dabei ist der Aspekt der Reziprozität zentral, unterscheidet er doch horizontale Solidarität von Fürsorge, die eine hierarchische und einseitige Form der Hilfe darstellt. Mit anderen solidarisch zu sein, bedeutet, davon auszugehen, dass die Rollen – wenn auch nur hypothetisch – getauscht sein könnten und man bei Bedarf ebenso mit Hilfe rechnen kann. Dies gilt auch für Formen der „Kampfsolidarität“ (ebd., S. 41), die durch das Verfolgen gemeinsamer Ziele gekennzeichnet sind.

In soziologischen Analysen findet der Begriff der Solidarität zwar vielfach Verwendung, ein Konsens zur Definition besteht jedoch nicht (Tranow 2012). So gibt es Vorschläge, Solidarität nur als emanzipatorischen politischen Begriff zu verwenden und für Fälle von Zusammenschlüssen derjenigen zu reservieren, die „Herrschafts- und Ungleichheitsverhältnissen“ unterworfen sind und nach Emanzipation streben (Scherr 2013, S. 264). Ein anderer, politisch-ethischer Debattenstrang fokussiert auf die Funktionsweisen von Solidargemeinschaften, Fragen nach der Reichweite des Kollektivs, der Belastbarkeit, der Verteilung von Ressourcen, des Einsatzes von Mitteln usw. Dem stehen Gebrauchsweisen gegenüber, die Solidarität eher deskriptiv in Anlehnung an Émile Durkheim als sozialen „Kitt“ verstehen, der für die Existenz sozialer Ordnung unerlässlich ist. Über diese Differenzen hinweg teilen die meisten Ansätze jedoch die Ansicht, dass Solidarität jenseits des Ideals universeller Solidarität mit der gesamten Menschheit in der Praxis stets sowohl Inklusion als auch Exklusion impliziert, da, folgt man etwa Smith und Sorrell (2014), das Einstehen für die Eigengruppe notwendigerweise mit dem Ausschluss von Fremdgruppen verbunden ist (Schnabel und Tranow 2020).

Der vorliegende Beitrag ist der zweiten genannten Orientierung zuzuordnen. Für unsere Studie erwies sich Steinar Stjernøs (2005) Zugang als gewinnbringend, der seinen Solidaritätsbegriff aus der Analyse unterschiedlicher politischer Programme europäischer Parteien seit dem 19. Jahrhundert entwickelt. Stjernø konzipiert Solidarität als ein Kontinuum, das sich zwischen dem Selbstbezug eines „Ich“ und seiner Identifikation mit allen anderen lebenden Menschen auf dem Planeten aufspannt: „The continuum of self and its identifications can be seen as moving from ‚I‘ to ‚All‘.“ (ebd., S. 16) Es geht also um das Verhältnis zwischen dem „Ich“ und dem „Wir“, wobei das „Wir“ sowohl kleinere Gruppen als auch größere soziale Gebilde meinen kann. Entlang dieses Kontinuums können auf der Grundlage von Exklusionsmechanismen – sprich: des Verhältnisses zwischen „wir“ und den „anderen“ –, anhand der Zielsetzungen von Gemeinschaften sowie des jeweiligen Verhältnisses von Individuum und Kollektiv unterschiedliche Konfigurationen von Solidarität differenziert werden (ebd., S. 16 ff.). Damit geraten die enge Verzahnung von Zugehörigkeitsgefühlen und daraus abgeleiteten Verpflichtungen zur gegenseitigen Unterstützung im Verhältnis zu sozialen Abgrenzungen und Exklusionsmechanismen in den Blick.

Von Bedeutung ist diese Perspektive insbesondere hinsichtlich des Wohlfahrtsstaats als einer speziellen Solidaritätskonfiguration. Mit der staatlichen Organisation der Solidargemeinschaft entstand ein rechtlicher Anspruch auf Unterstützung im Notfall, wodurch die Abhängigkeit von freiwilliger Wohltätigkeit überwunden wurde. Die Unterstützung erfolgt nicht mehr persönlich, sondern ist in Versicherungsleistungen oder Beihilfen institutionalisiert (Bayertz 1998, S. 37; Laitinen und Pessi 2015, S. 9; Scholz 2008). Der Wohlfahrtsstaat fordistischer Prägung beruht dabei sowohl auf einer „Logik der Nation“ als auch einer „Logik des Produktivismus“ (Lessenich 2006, S. 182) und produziert entlang dieser Linien systematisch Ausschlüsse (z. B. von Zugewanderten oder Nicht-Erwerbstätigen), indem die Reichweite der Solidarität nach nationalstaatlichen Grenzen und nach der Bereitschaft und Fähigkeit zur Beitragsleistung und der „Würdigkeit“ der Hilfsbedürftigen gezogen werden (vgl. u.a. van Oorschot 2000).^4^ Nicht nur im Hinblick auf ihre Entstehung ist die institutionalisierte Solidarität des Sozialstaates eng mit den Interessen gesellschaftlicher Klassen und den Kämpfen zwischen diesen verbunden. Auch die Haltungen der Bürger*innen zu den aktuellen Auseinandersetzungen um den Rückbau, Erhalt oder Ausbau des Sozialstaates sind nicht unabhängig von ihrer Klassenlage zu verstehen.

### Klasse: Identifikation und Distinktion

Gipfelnd in Ulrich Becks (1994, S. 52) Verkündung des Endes der Klassengesellschaft, galt Klasse als analytisches Konzept in der deutschsprachigen Soziologie seit den 1980er-Jahren lange als überholt. Im Zuge von Modernisierungsprozessen seien, so Becks These, die Grundpfeiler der Industriegesellschaft und damit auch die Wirkung von Klasse, aber auch Geschlecht oder Familienbindungen, brüchig geworden. Den Bedeutungsverlust von Klasse verortete Beck sowohl auf der sozialstrukturellen Ebene, da Ungleichheitslagen nicht mehr entlang von sozialer Herkunft bestimmt würden, als auch auf der subjektiven Ebene, weil „die lebensweltliche Identität sozialer Klassen“ (ebd.) weggeschmolzen sei und es entsprechend an Klassenbewusstsein fehle.

Entgegen dieser vor allem im deutschsprachigen Raum populären These vom „Ende der Klassengesellschaft“ lassen sich seit der Jahrtausendwende verschiedenste Bemühungen beobachten, neben den rein quantitativ vorgehenden Sozialstrukturanalysen auch die qualitative Klassenanalyse neu zu beleben (Vester et al. 2001; Dörre et al. 2013; Altreiter 2019). Dabei wird insbesondere auf das Klassenverständnis von Pierre Bourdieu (1987a, b, S. 171 ff.) Bezug genommen, demzufolge sich Gesellschaft als mehrdimensionaler „sozialer Raum“ konzipieren lässt, in dem die Gesellschaftsmitglieder auf der Grundlage der ihnen verfügbaren Kapitalien relational zueinander positioniert sind. Im Sinne Bourdieus sind „Klassen“ demzufolge gleichbedeutend mit Gruppen von Personen, die sowohl hinsichtlich ihrer objektiven sozialen Lage aufgrund ökonomische und kulturelle Kapitalien als auch geteilter Praktiken, Denk- und Wahrnehmungsformen eine Homogenität aufweisen. Eindeutige Grenzen zwischen verschiedenen Klassen sind in diesem Verständnis schwer auszumachen, es handelt sich eher um grob abgegrenzte Cluster. Bourdieu (1998, S. 23; 1985) verweist selbst darauf, dass es sich bei „Klassen“ in seiner Theorie um Konstrukte handelt, weshalb er auch von „theoretischen Klassen“ spricht, die eine „größtmögliche Homogenität“ im Hinblick auf Kapitalausstattung und Lebensführung aufweisen. Demensprechend teilen die zugeordneten Individuen auch nicht automatisch ein politisch mobilisierbares „Klassenbewusstsein“. Bourdieu (1987b, S. 738) spricht stattdessen vom „Habitus“ als latenter, unbewusster Manifestation der Position im sozialen Raum, der die Akteur*innen mit einem „sozialen Orientierungssinn“ dafür ausstattet, wo sie selbst im Verhältnis zu anderen im sozialen Gefüge situiert sind.

Zwei Anmerkungen sind an dieser Stelle notwendig. Erstens unterscheidet sich der Zugang von Bourdieu mit der systematischen Verbindung von Position und Disposition fundamental von der Lebensstilforschung in der Tradition von Gerhard Schulze u.a., in der – mit Karl Marx gesprochen – „Sein“ und „Bewusstsein“ nicht mehr länger miteinander verbunden sind. Entsprechend divergiert auch das Verständnis von Milieus, das in beiden Theorietraditionen gebräuchlich ist: Spricht Schulze (1992, S. 23) von Milieus als „große[n] Personengruppen mit ähnlichen subjektiven und situativen Merkmalen, die sich voneinander durch erhöhte Binnenkommunikation abheben“, so verwendet Bourdieu den Milieu-Begriff zum Teil synonym zum Klassenbegriff, typischerweise aber als Bezeichnung für kleinere Segmente einer Klassenlage. Zweitens haben wir es bei Bourdieu, wie auch bei Marx oder Weber, mit einem *relationalen* Verständnis von Gesellschaft zu tun (vgl. u.a. Wright 1985, S. 58). Im Gegensatz zur klassischen Schichtforschung, die verschiedene Schichten auf Basis individueller Attribute von Individuen konstruiert und nicht zueinander in Beziehung setzt, geht Bourdieu davon aus, dass Menschen über Machtbeziehungen miteinander verbunden sind, diese Beziehungen jedoch zugleich Gegenstand konstanter Positionskämpfe sind. Die Ursachen sozialer Ungleichheit liegen in einem Abgrenzungs- *und* Abhängigkeitsverhältnis sozialer Gruppen, das auf Ausbeutung und dem Ausschluss nicht-privilegierter bzw. beherrschter Klassen vom Zugang zu Ressourcen fußt.

Für das hier verfolgte Anliegen der Untersuchung des Nexus von Klassenlage und Solidaritätskonfiguration sind insbesondere Bourdieus Hinweise bedeutsam, dass die Angehörigen der oberen Klassenlagen im Kampf um die Reproduktion der objektiven Kräfteverhältnisse versuchen, mittels komplexer Distinktionsprozesse soziale und ökonomische Chancen zu monopolisieren. In diesem Prozess der „sozialen Schließung“ (Weber 1985, S. 184) spielt neben der Kapitalausstattung nicht zuletzt der bereits genannte Habitus eine zentrale Rolle. Als „praktischer Operator“, der „objektiv klassifizierte Praxisformen […] in klassifizierende, d. h. in einen symbolischen Ausdruck der Klassenstellung [verwandelt]“ (Bourdieu 1987b, S. 284), ermöglicht es etwa der habituelle „Geschmack“ den herrschenden Klassenlagen, die eigene Lebens- und Deutungsweise als überlegen, damit als „legitim“ im Unterschied zum Habitus der Angehörigen beherrschter Klassenlagen zu markieren. Symbolische Grenzziehungen verstärken die sozialen Grenzen zwischen den Klassenlagen, ja machen diese überhaupt erst erfassbar (ebd., S. 278). Darüber hinaus reproduzieren sie Trennungen nach ethnischer Zugehörigkeit und behindern dadurch in den unteren Klassenlagen das Erkennen sowie das gemeinsame Handeln im Sinne geteilter Interessen.

Der symbolischen Ebene kommt in Bourdieus Klassenanalyse mithin eine zentrale Bedeutung zu. Im Anschluss daran wurde unter dem Label der „symbolic boundary“-Forschung in den letzten Jahren eine Vielzahl von Arbeiten publiziert, die sich einer Weiterentwicklung dieses Aspekts der Bourdieu’schen Theorie widmen (vgl. dazu u.a. Lamont und Molnár 2002). Verantwortlich für die Popularisierung des Begriffs der „symbolischen Grenzziehung“ zeichnet dabei Michèle Lamont (1992). In Anlehnung an Bourdieu wird in dieser Forschung auf kulturelle, moralische und sozio-ökonomische Attribute von Praktiken der Abgrenzung geachtet und betont, dass Personen die Tugenden, die sie sich selbst zuschreiben (z.B. Fleiß, Bodenständigkeit), üblicherweise als universellen Maßstab zur moralischen Bewertung ihrer Mitmenschen gebrauchen (Lamont 2000; Sayer 2005, S. 184). So zeigen beispielsweise Studien von Patrick Sachweh in Deutschland, dass Angehörige unterer Klassenlagen sich selbst für solidarischer halten als Angehörige höherer Klassenlagen, die sie eher als rücksichtslos und egoistisch bewerten (Sachweh 2013, S. 23). Bei symbolischen Grenzziehungen handelt es sich um Klassifikationspraktiken sozialer Akteur*innen, in deren Verlauf relational die Praktiken, Geschmäcker und Werthaltungen anderer Gesellschaftsmitglieder kategorisiert werden und die daher (Ab‑)Wertungen und soziale Schließungen vollziehen.

Um die Verbindungen von Klassenlage und Solidaritätskonfigurationen zu untersuchen, rekurrieren wir im Folgenden ausgehend von Bourdieu auf insgesamt drei analytische Dimensionen des Klassenbegriffs: Erstens begreifen wir „Klasse“ als objektive soziale Lage im Sinne einer Positionierung im sozialen Raum und der Verfügbarkeit von Machtressourcen; zweitens nehmen wir „Klasse“ im Sinne eines Klassenhabitus in den Blick, der latente Zugehörigkeiten und Identifikationen (auch im Sinne positiver Selbstzuschreibungen), aber damit notwendigerweise auch Distinktionspraktiken in Form symbolischer Grenzziehungen gegenüber anderen hervorbringt. Darüber hinaus fragen wir drittens danach, ob es auch Hinweise auf ein manifestes Klassenbewusstsein gibt, das Zugehörigkeiten und Interessen reflektiert und benennt. Alle drei Ebenen werden berücksichtigt, wenn es im Folgenden darum geht auszuloten, welchen Beitrag ein Blick auf Klassenlagen und Klassenhabitus im Sinne Bourdieus für das Verstehen von Solidaritätskonfigurationen und insbesondere der Haltungen zur institutionalisierten Solidarität des Sozialstaates leistet.

### Typologie der Solidaritäten

Ausgehend von Stjernøs Kontinuum der Solidaritäten, das sich zwischen idealtypischen Extrempolen aufspannt, haben wir zunächst dessen vorgeschlagene Dimensionen auf das empirische Material angewandt und die Heuristik daraufhin auf insgesamt fünf Dimensionen ausgeweitet. Übernommen haben wir die Dimensionen der Identifikation (*Zugehörigkeit*) und des Inklusionsgrads (*Reichweite*), die beide auf den Umfang der Solidargemeinschaft verweisen. Im Zentrum steht dabei die Frage, mit welchen sozialen Gruppen sich Menschen verbunden fühlen – wen sie also meinen, wenn sie von „wir“ sprechen (z.B. Benachteiligte und Unterdrückte, Österreicher*innen, Leistungsträger*innen) – und wer als Teil der Solidargemeinschaft wahrgenommen wird. Hinzugefügt haben wir darüber hinaus die Dimension der *Bedingungen*, die Aussagen in der Richtung umfasst, ob und in welcher Weise Solidarität an bestimmte Erwartungen oder Gegenleistungen geknüpft ist (z.B. Tugendhaftigkeit, Dankbarkeit, Aufnahme einer Erwerbsarbeit, Anpassung an die Mehrheitsgesellschaft). Als vierte Dimension haben wir die *Gerechtigkeitsprinzipien *ergänzt, die für das Verständnis der Entstehung moralischer Verpflichtungen zur Unterstützung zentral sind und die Frage betreffen, mit welchen Kriterien die Solidaritätsbereitschaft assoziiert wird (z.B. Leistung, Gleichheit, Bedarf oder auch Status). Als fünfte Dimension fassen wir schließlich *Aktivität*, also die Frage, ob moralische (Selbst‑)Verpflichtung zu eigenem solidarischen Handeln motiviert (z.B. in Form von ehrenamtlichem Engagement, politischem Aktivismus etc.) oder Handlungserwartungen eher an Dritte – nicht zuletzt: an den Sozialstaat – gerichtet werden.

Entsprechend den Ausprägungen entlang dieser Dimensionen haben wir ähnliche Fälle zu jeweils einem Typus gebündelt. Anleitend für die Typenbildung war ein möglichst hohes Maß an interner Homogenität im Hinblick auf die Solidaritätsorientierungen, bei gleichzeitig größtmöglicher Varianz zwischen den Typen (Kelle und Klug 2010, S. 112). Zugleich sind Überlegungen zur Bildung von „Idealtypen“ im Sinne Webers zum Tragen gekommen, insofern bewusst „einseitige Steigerung[en] eines oder einiger Gesichtspunkte“ (Weber 1985, S. 190) vorgenommen wurden, um charakteristische Aspekte der Typen zu konturieren. Zugleich sind wir uns bewusst, dass kein Fall mit einem Typus „ident“ sein kann, auch wenn die Typenbildung notwendig am individuellen Fall ansetzt (Przyborski und Wohlrab-Sahr 2014, S. 380). Es handelt sich vielmehr bei jedem Typus um eine „theoretische Verdichtung“ (ebd.), die nicht alle Facetten eines Falles enthalten kann und je nach inhaltlicher Fokussierung auch anders erfolgen könnte.

Die Auswertung der Interviews ergab sieben verschiedene Solidaritätstypen, die sich durch die jeweiligen Vorstellungen von der Gesellschaft, die empfundenen Zugehörigkeiten zu einzelnen Gruppen ebenso wie durch die Grenzziehungen, die sie um die Solidargemeinschaft vornehmen, und die Bedingungen, die sie für eine Aufnahme (bzw. einen Verbleib) stellen, unterscheiden. Darüber hinaus ziehen die dargestellten Typen aus ihren Wahrnehmungen und Deutungen unterschiedliche Konsequenzen im Hinblick auf ihr eigenes (solidarisches) Handeln und ihre Ansprüche auf solidarischen Beistand. Zwar war die Klassenlage der Befragten nicht unmittelbar Gegenstand der Typenbildung; jedoch wirkte das Bourdieu’sche Klassenverständnis, das von einer Homologie von sozialer Lage und individuellen Denk‑, Wahrnehmungs- und Handlungsweisen ausgeht, informierend bei der Interpretation der Daten und dem Verständnis von Teilaspekten der Solidaritätskonfigurationen. Die „objektive“ ökonomische Klassenlage wurde mithilfe des sozialstatistischen Fragebogens und Hinweisen aus den Interviews rekonstruiert und entsprechend mit den Deutungsweisen der Befragten kontextualisiert.

Die Vorstellung von Solidarität als Kontinuum hat sich als sinnvoll erwiesen, um die Komplexität sozialer Ein- und Ausschlüsse zu fassen. Die darauf aufbauende Unterscheidung der fünf Dimensionen macht nachvollziehbar, wie divergierende Inklusions- und Exklusionsmechanismen miteinander verbunden sind. Das Kontinuum ist daher weniger als lineare Achse denn als multidimensionaler Raum zu verstehen. Wir können zwar von Typ 1 zu Typ 7 zunächst eine Abnahme der Reichweite der Solidarität feststellen, insofern zunehmend leistungsbezogene, nationale und ethnische Grenzen eingezogen bzw. Bedingungen gestellt werden. Gleichzeitig lassen sich beispielsweise die auf bestimmte Gerechtigkeitsvorstellungen bezogenen Grenzziehungen nicht eindeutig nebeneinander aufschlüsseln und sind eher qualitativ als quantitativ unterscheidbar. Zwischen den Polen spannt sich also ein breites Spektrum an Solidaritätskonfigurationen unterschiedlichen Umfangs auf (Tab. 2).

**Table 2:** 

*Solidaritätsorientierung* *(Zahl der Befragten)*	*Soziales Profil*	Solidaritätsdimensionen				
		*Zugehörigkeit*	*Reichweite*	*Bedingungen*	*Gerechtigkeits-prinzip*	*Aktivität*
**Politisch (6)**	Urbane Gebildete in prekärer Erwerbsarbeit	Weltgesellschaft	Bedürftige (alle)	Keine	Gleichheitsprinzip	Zivilgesellschaftliches und gesellschaftspolitisches Engagement
**Altruistisch (9)**	Urbane Privilegierte	Weltgesellschaft	Bedürftige (alle)	Keine	Bedarfsprinzip	Zivilgesellschaftliches und karitatives Engagement
**Ermöglichend leistungsorientiert (8)**	Rurale und kleinstädtische gehobene Dienstleistungsklasse und Unternehmertum	Erwerbstätige Bevölkerung	Bedürftige Leistungsbereite (alle)	Leistungswille, Bereitschaft zur Erwerbsarbeit	Bedarfs- und Leistungsprinzip („Fördern und Fordern“)	Zivilgesellschaftliches und karitatives Engagement
**Beitrags-** **orientiert (7)**	Rurales Handwerks- und Arbeiter*innenmilieu	Erwerbstätige Bevölkerung	Bedürftige Leistungsbereite (alle)	Leistungswille, Bereitschaft zur Erwerbsarbeit	Leistungsprinzip	Keine
**Moralisierend autoritär (7)**	Rurales und kleinstädtisches Kleinbürgertum	Erwerbstätige Bevölkerung	Stark bedürftige Leistungs- und Anpassungsbereite (alle)	moralisch richtige Lebensführung, Leistungswille	Bedarfs- und Leistungsprinzip	Keine
**National ausgrenzend (3)**	Rurales Handwerksmilieu; kleinstädtische Dienstleistungsklasse	Erwerbstätige Bevölkerung, nationales Kollektiv	Bedürftige Leistungsbereite (national)	Leistungswille, Bereitschaft zur Erwerbsarbeit	Status- und (nationales) Bedarfsprinzip	Keine
**Ethnonational ausgrenzend (3)**	Kleinstädtisches Klein-bürgertum	Ethno- nationales Kollektiv	Bedürftige Leistungsbereite (ethnisch-national)	Ethnische Herkunft, moralisch richtige Lebensführung und Leistungswille	Statusprinzip	Fallweise parteipolitisches Engagement

Quelle: Eigene Darstellung

## Die sieben Solidaritätstypen

Die nachstehende Analyse fokussiert auf einen Teilaspekt der Studie, und zwar auf die sozialstaatsbezogenen Solidaritätsorientierungen, die sich in den Interviews insbesondere in der Auseinandersetzung mit zwei sozialen Gruppen – nämlich erwerbslosen Personen und Geflüchteten bzw. Zugewanderten – manifestiert. Im Folgenden werden zunächst jeweils die charakteristischen Positionierungen der einzelnen Typen im Hinblick auf den Sozialstaat skizziert und im Anschluss jeweils gezeigt, inwiefern die Berücksichtigung der Klassenlage es erleichtert, die jeweilige Solidaritätskonfiguration zu verstehen und zu erklären.

### Politische Solidarität (Typ 1)^5^

Charakteristisch für diesen Typus ist ein ambivalentes Verhältnis zu staatlicher Autorität und Sozialstaatlichkeit. So sind die diesem Typ zugeordneten Interviewten der Meinung, dass der Staat die Aufgabe hat, den Schutz und die Versorgung von nicht-privilegierten Bevölkerungsgruppen zu gewährleisten. Es sei Aufgabe des Staates, „gewisse Rechte zu gewährleisten und zu schützen, die ohne sein Eingreifen im Lauf der Dinge untergehen würden“ (Int_19), und „alle mit den gleichen Sozialleistungen zu versorgen“ (Int_18). Gleichzeitig bleibt jedoch eine Grundskepsis gegenüber dem Sozialstaat und staatlicher Autorität spürbar. Ein Grund dafür sind eigene Erfahrungen, dass der Staat seiner Aufgabe, die Schwächsten zu schützen, nicht ausreichend nachkommt bzw. sogar gegenteilig verfährt. Johanna Dörfler und Sarah Eder beispielweise berichten, dass sie während der Erwerbsarbeitslosigkeit vom Arbeitsmarktservice^6^ nicht unterstützt, sondern unter Druck gesetzt werden:

Als Außenstehender denkst du dir: Ach Gott, das sind nette Menschen, die helfen dir, wieder einen Job zu finden. [...] das war ein ziemlich herbes Aufwachen. (Int_09)

Ich habe ihm [dem Kundenbetreuer, d. A.] dann auch geschrieben und habe ihm gesagt: „Ich habe in meinem Leben wirklich sehr viel gearbeitet und eben auch viel unbezahlt. Und ich bin Mutter von drei Kindern, und auch das ist ein Job, ja? Und ich wünsche mir einfach den Respekt“, also, dass auch er mir den Respekt zollt, der mir gebührt. (Int_07)

Auch während der Fluchtbewegungen im Jahr 2015 wurde der Staat als unzulänglich erlebt, die Situation konnte in den Augen der Befragten nur durch den unermüdlichen Einsatz der Zivilgesellschaft bewältigt werden.

Charakteristisch für diesen Typus ist ein dualistisches Gesellschaftsbild, in dem sich zwei soziale Gruppen antagonistisch gegenüberstehen: Herrschende und Beherrschte, Privilegierte und Nicht-Privilegierte: „Da gibt es“, so Sarah Eder, „auf der einen Seite Menschen, die 40 Stunden arbeiten oder aus einem zerbombten Land kommen und im Elend sitzen. Und dann gibt es den Chef von der Post, der mit 800.000 Euro im Jahr heimgeht dafür, dass er die Leute quält.“ (Int_09) Dieser Antagonismus bleibt nicht auf den Nationalstaat begrenzt, sondern wird als globales Strukturierungsprinzip gedeutet: „Wenn ich das Wort ‚Wirtschaftsflüchtlinge‘ höre, kommt mir die Galle hoch, weil: Schauen Sie sich einmal an, wie Leute in anderen Ländern für unsere Konsumgesellschaft arbeiten und unter welchen Bedingungen.“ (ebd.) Die Befragten schöpfen einen zentralen Teil ihrer Identität aus dem Zugehörigkeitsgefühl zur globalen Klasse der Entrechteten. Unterdrückung und Ausbeutung begreifen sie als immanente Eigenschaften eines Systems, dem man in einem gemeinsamen Kampf begegnen müsse: „Es geht uns einfach allen besser, wenn wir solidarisch sind. Ja, und ich glaube, man müsste es sogar global irgendwie sehen“ (Int_07).

Christoph Lehner argumentiert mit Blick auf die Flüchtlingshilfe im Jahr 2015, dass das Engagement für die Anliegen von Geflüchteten kein Einsatz für „die anderen“ sei. In diesem Sinne kritisiert er die vielen Helfenden: „Die Hilfe, die man leistet, die Dinge, für die man sich einsetzt, sind dann immer nur für die anderen, ist aber nie etwas, das einen selbst betrifft.“ (Int_19) Dies führe zu einer „Retterhaltung“, in der man den Betroffenen nicht auf „Augenhöhe“ begegnet. Der Einsatz für die Ausweitung sozialstaatlicher Unterstützung und angemessener Wohnversorgung oder für den Zugang zu Bildung ist im Verständnis dieser Gruppe von Befragten Teil eines Kampfes für die Verbesserung der Chancen *aller* Nicht-Privilegierten einer Gesellschaft, nicht nur von Geflüchteten. Bei den Interviews mit dieser Gruppe fiel zudem auf, dass ihre Solidarität nicht nur über nationalstaatliche Grenzen reicht, sondern auch bedingungslos ist. Für die Unterstützung anderer werden keine Voraussetzungen formuliert, die diese zu erfüllen hätten. Ebenso wenig wird von ihnen eine Gegenleistung erwartet.

Welche Aspekte von Klassenlagen ermöglichen es zu verstehen, wie diese spezifische Solidaritätskonfiguration zustande gekommen ist? Die Befragten gehören urbanen Milieus an, sie verfügen über Matura oder haben einen Hochschulabschluss erworben. Zugleich teilen sie mehrheitlich eine prekäre Stellung auf dem Arbeitsmarkt, die jedoch unterschiedlich begründet ist: einerseits durch prekäre Übergänge in den Arbeitsmarkt während bzw. nach dem Studium (vorübergehende Prekarität) und andererseits durch langjährige Beschäftigung im Niedriglohnsektor und Phasen der Arbeitslosigkeit (verfestigte Prekarität). Durch Selbstzuordnung zu einer globalen Klasse der Unterdrückten weisen die Befragten ein Klassenbewusstsein ohne nationale Begrenzungen auf. Gleichzeitig gibt es unter ihnen ein ausgeprägtes Bewusstsein dafür, dass es auch unter Nicht-Privilegierten erhebliche Unterschiede gibt, was finanzielle Mittel oder politische Mitbestimmungsmöglichkeiten betrifft.

Während sie mithin insgesamt eine große Sensibilität für verschiedene Ungleichheitsrelationen zeigen, gelingt es den Befragten gleichzeitig, unterschiedlichste Anliegen auf sich selbst zu beziehen und dadurch ihr Zugehörigkeitsgefühl und somit die Konstruktion eines „Wir“ zu bestärken. Es herrscht hier die Überzeugung vor, dass die Rechtlosen nur dann zu ihrem Recht kommen, wenn sie füreinander einstehen, weil man nur „gemeinsam etwas bewirken kann“ (Int_09). Empörung macht sich dann breit, wenn die Entrechteten gegeneinander „ausgespielt“ (Int_05) werden. Die Solidarität mit anderen spiegelt sich in einem hohen Ausmaß an gesellschaftspolitischem Engagement. Christoph Lehner und Mario Lenz beraten Geflüchtete in Asylrechtsfragen, Johanna Dörfler und Sarah Eder sind Mitglieder in einer Erwerbslosengruppe, und Richard Berger ist in einem Verein aktiv, der sich für eine gerechte Vermögensverteilung einsetzt. Aber auch politisch ist diese Gruppe im Vergleich zu den anderen Typen am aktivsten. Das reicht von selbstorganisierten Gruppen im politisch linken Spektrum bis zur Mitgliedschaft in Sektionen der Sozialdemokratischen Partei.

Es treffen sich in diesem Typus zwei verschiedene Gruppen im sozialen Raum. Zwar verfügen beide über sehr wenig ökonomisches Kapital, jedoch sind die einen nach einem Abstieg von manifester Armut betroffen, während die anderen nach dem Studienabschluss sehr niedrige Einkommen haben, aber durch die privilegierte Stellung der Eltern auf materielle Absicherung zählen können. Gemeinsam ist beiden Gruppen ein hohes Ausmaß an kulturellem Kapital, insbesondere auch an politischer Bildung und Praxis. Dies lässt insgesamt die gemeinsame kampforientierte Solidaritätskonfiguration verständlich werden.

### Altruistische Solidarität (Typ 2)^7^

Der Sozialstaat gerät bei diesen Befragten in erster Linie in seiner Funktion als Unterstützer für Bedürftige und für die „Ärmsten“ (Int_16) in den Blick. Die Repräsentant*innen dieses Typs gehen grundsätzlich davon aus, dass es in Österreich einen gut funktionierenden Sozialstaat gibt, auch wenn es noch „Verbesserungsmöglichkeiten geben würde“ (Int_23). Es gibt die Gewissheit, „dass Menschen, die in Not geraten, ein Netz haben, wo sie aufgegangen werden“. Auch wenn nicht alles so „toll“ ist, „es wird einem geholfen, und es wird irgendwie geschaut, dass man mit seinen Schulden zurechtkommt, und man kann irgendwie existieren, und dann kann man wohin gehen und Lebensmittel holen, wo man nichts zahlen muss“ (ebd.). Einigkeit herrscht darüber, dass in Österreich Menschen, die aus verschiedensten Gründen in Not geraten sind, sozialstaatlich geholfen werden muss – und zwar unabhängig davon, ob sie zuvor Beiträge in das Sozialsystem einbezahlt haben oder nicht. Kürzungen von Sozialleistungen lehnen diese Befragten deshalb durchweg ab.

Die Befragten nehmen sich selbst als Privilegierte in der Gesellschaft wahr und leiten daraus eine Verpflichtung zur Solidarität ab. In der Literatur wird diese Motivlage auch als „guilt over affluence“ (Bierhoff 2002, S. 155) beschrieben. So begründet Sophie Billinger ihr Engagement in der Flüchtlingsbetreuung damit, dass ihr bewusst sei, „wie gut es meiner Familie geht, und ich sage, man ist der Menschheit was schuldig, wenn es einem gut geht“ (Int_06). Die solidarische Haltung ist in dieser Gruppe jedoch nicht nur der eigenen privilegierten Stellung geschuldet, sondern auch durch ein humanistisches Wertefundament motiviert, das bei einigen durch Hinweise auf universelle Menschenrechte, bei anderen durch christliche Werthaltungen fundiert ist. Die Personen, die wir diesem Typ zugeordnet haben, leiten daraus die Verpflichtung ab, erstens durch höhere Steuern ihren Beitrag zum Sozialstaat zu leisten und zweitens Geflüchteten aktiv zu helfen, weil der Staat in der Frage versagt habe. Vor diesem Hintergrund wird eine Steuerpolitik, die Besserverdienenden einen höheren Beitrag abverlangt, dezidiert befürwortet: „Ich“, so Barbara Pollak, „find’s gut, dass wir hohe Steuern haben“, auch wenn sie „manchmal schluckt“ (Int_23). Obwohl es eine breite Zustimmung zu dieser Umverteilung von Ressourcen gibt, wird dennoch eine Begrenzung des Ausmaßes dieses Beitrags erwartet, wie das Zitat des Arztes Josef Klein deutlich macht: „Ich glaube, es ist nicht sinnvoll, wenn man die Steuern wesentlich höher als 50 % einbetoniert. Also in Schweden war das ja, glaube ich, bis 70 % oder so. Da hört sich dann der Spaß bald auf. Da fühlen sich diese Menschen [die Besserverdienenden, d. A.] wieder ausgenützt.“ (Int_16)

Die subjektive Wahrnehmung, privilegiert zu sein, korrespondiert auch auf der objektiven Ebene mit einem überdurchschnittlichen Ausmaß an kulturellem und bei den älteren Befragten auch ökonomischem Kapital. Alle Befragten haben Matura, die Mehrheit einen Hochschulabschluss, ihr Haushaltsnettoeinkommen liegt zwischen 4000 und 7000 Euro. Die Gruppe befindet sich in sicherer Beschäftigung und fühlt sich mehrheitlich beruflich anerkannt. Darüber hinaus ist die gute Position durch Immobilienbesitz, wie Eigentumswohnungen und Wochenendhäuser, abgesichert. Die Angehörigen dieser Gruppe entstammen überwiegend Familien der oberen Mittelschicht, wobei die älteren Befragten ihre Herkunftsprivilegien durch den eigenen beruflichen Werdegang noch ausbauen konnten. Existenzielle Unsicherheiten gehören nicht zu ihren Erfahrungen, wie das Zitat von Evelyn Rauter zeigt: „Weder habe ich Angst um meinen Job, noch habe ich Angst, dass ich keinen finde, […] wenn ich meinen verliere. Und ich habe Eltern, die mich unterstützen. Ich bin halt einfach abgesichert.“ (Int_25)

Aus der Herkunft resultiert eine große Distanz zu den Lebenswelten der Nicht-Privilegierten – wobei der Umstand, dass sie sozial „abgeschottet“ sind, diesen Personen zum Teil durchaus bewusst ist. So spricht Josef Klein von der „geschützten Werkstätte meines Lebens“ (Int_16), die keine Einblicke in soziale Randlagen in Österreich ermöglicht habe. Ingrid Kramer erzählt von sozialen Projekten in der Privatschule ihrer Kinder, damit die Kinder auch „Obdachlose“ kennenlernen (Int_17). Diese soziale Distanz zu weniger privilegierten Klassen ist auch der Grund dafür, dass soziale Ungleichheitslagen innerhalb Österreichs im Bewusstsein dieser Gruppe marginal sind. Ähnlich wie Michael Vester (2017) für das radikaldemokratische Lager beschreibt, scheinen auch hier vor allem neue soziale Ungleichheiten, wie Geschlecht oder Ethnizität, präsent. Das Bewusstsein für „alte“, klassenbezogene Ungleichheiten fehlt hingegen großenteils. Dementsprechend entzündete sich die Empörung während der großen Fluchtmigration im Jahr 2015 an der unmittelbar evidenten Diskrepanz zwischen dem eigenen Status als Privilegierte und den als benachteiligt wahrgenommenen Geflüchteten. Das Leid bedürftiger Menschen, die schon lange in Österreich leben, erregt demgegenüber vergleichsweise wenig Handlungsimpulse: Darum solle sich der Staat kümmern, der das bislang gut gemacht habe. Kritik und emotionale Aufregung, wie sie bei Erzählungen zum Umgang mit Geflüchteten deutlich zu spüren ist, fehlt, wenn es um verfestigte soziale Randlagen in Österreich geht.

Wie auch jene mit politischer Solidaritätsorientierung waren die Befragten dieses Typs nach 2015 stark in der Flüchtlingshilfe engagiert. Während sie im Hinblick auf inländische soziale Randlagen dem Staat vertrauen, entsprechend Verantwortung zu übernehmen, sind sie hier bereit, ergänzend zum Sozialstaat – der vielfach als ungenügend erfahren wird, etwa was Wohnungssuche, Nachhilfe oder Amtswege betrifft – unterstützend einzugreifen. Allerdings ist diese Hilfe im Gegensatz zum Typ 1 eher als altruistisch denn als solidarisch zu bezeichnen. Helfende und Empfänger*innen der Hilfe sind nicht gleichgestellt, man sieht sich nicht als Teil einer Gruppe, die für gemeinsame Anliegen kämpft. Die Hilfe ist für *andere* gedacht, wodurch die Grenzen zu paternalistischen Haltungen gelegentlich verschwimmen. Der Gedanke, selbst bedürftig zu werden und vor allem im ökonomischen Sinn auf andere oder den Staat angewiesen zu sein, liegt außerhalb ihrer Vorstellung und ist aufgrund ihrer sozialen Lage tatsächlich sehr unwahrscheinlich.

Insgesamt wird in diesem Typus Ungleichheit in der Gesellschaft nicht grundsätzlich in Frage gestellt, auch wenn sie zu eigenen Unterstützungsleistungen anregt. Es geht eher darum, ein zu großes Ausmaß an Ungerechtigkeit zu reduzieren, ohne dabei die eigene privilegierte Stellung zu gefährden.

### Ermöglichende leistungsorientierte Solidarität (Typ 3)

Auch die Befragten dieses Typs äußern sich sehr positiv gegenüber einem „gut ausgebauten“ Sozialstaat. Sie haben die Bandbreite sozialstaatlicher Leistungen von der Sozialversicherung über das Arbeitslosengeld bis zur Sozialhilfe und der Betreuung obdachloser Personen oder Geflüchteter im Blick und lehnen eine Kürzung dieser Leistungen ab. Ausreichende Transferzahlungen sind aus ihrer Sicht unter anderem auch deshalb notwendig, damit Menschen ohne oder mit zu niedrigem Einkommen nicht aus Verzweiflung kriminell werden und die öffentliche Sicherheit gefährden. Sascha Baumann etwa begründet seine Ablehnung von Kürzungen im Sozialbereich damit, dass er seinen „sozialen Frieden“ haben möchte, „ich will aber nicht, dass man dich jetzt auf 400 Euro kürzt, und du bist verzweifelt und gehst einbrechen“ (Int_03). Auch Hans Niedermoser meint, „dass man nicht immer weiter nach oben verteilen kann, weil das einfach brandgefährlich ist, wenn die im unteren Bereich dann schon drei Jobs haben und es geht sich noch immer nicht aus mit Miete und Essen“ (Int_21).

Im Unterschied zu den bisher dargestellten Solidaritätskonfigurationen werden den Empfänger*innen von staatlichen Unterstützungsleistungen Bedingungen für die Gewährung dieser Hilfe gestellt: Die Befragten heben das „Geben und Nehmen“ hervor. „Alle, die können, müssen einen Beitrag leisten“, um, so die Befragte Sabine Friedrich, „das bestehende Sozialsystem aufrechtzuerhalten“ (Int_12). Dieser Beitrag wird v. a. in Form einer Bereitschaft zur Erwerbsarbeit erwartet. Für alle, die arbeiten können, gilt die Verpflichtung zur Erwerbsarbeit. „Wenn man einem ein Zuckerl gibt, kann man etwas einfordern“, meint beispielweise Manfred Rabl im Hinblick auf sozialstaatliche Hilfen, der „Sozialstaat ist kein Polster, auf dem man sich ausruhen darf“ (Int_24). Dabei haben diese Personen durchaus im Blick, dass es nicht allen Gesellschaftsmitgliedern möglich ist, diese Verpflichtung zu erfüllen. Deshalb betonten sie die Verpflichtung der Gemeinschaft, die Bemühungen der Bedürftigen zu unterstützen, sich in die Lage zu versetzen, einen Beitrag leisten zu können. Hier finden wir Vorschläge in Richtung einer aktivierenden Sozialpolitik. Das ist aus Sicht dieser Befragten mit arbeitsmarktpolitischen Förderprogrammen, etwa zur Qualifizierung oder der Unterstützung beim Schritt in die selbstständige Erwerbstätigkeit, möglich. Der Unternehmer Hans Weis fasst seine Haltung so zusammen:

Mein Ziel wäre, egal ob das jetzt Migranten oder sozial benachteiligte Menschen sind, sie zu ermächtigen, ihnen Selbstvertrauen zu geben durch Bildung, durch Zuwendung. Zu sagen: „Komm’ dir nicht wie ein Würsterl vor. Ich sehe dich nicht so. Ich sehe dein Potenzial. Mach’ deine – keine Ahnung – Firma auf. […] Und wenn du es nicht kannst, wir versuchen, dich zu unterstützen.“ (Int_30)

Insgesamt enthält die Solidaritätskonfiguration im Hinblick auf den Sozialstaat also ein differenziertes Gefüge des „Förderns und Forderns“^8^. Die Solidargemeinschaft des Sozialstaates muss bei Bedarf alle unterstützen können. Damit dieses Arrangement aber möglich ist, erwächst allen Mitgliedern eine zweifache Verpflichtung: Erstens müssen sie über Erwerbstätigkeit zur Aufrechterhaltung dieser Solidargemeinschaft beitragen. Wenn sie in eine Situation geraten, durch die sie auf Unterstützung durch den Sozialstaat angewiesen sind, haben sie – solange sie im erwerbsfähigen Alter und nicht aufgrund von Krankheit erwerbsunfähig sind – zweitens die Pflicht, sich zu bemühen, sich aus dieser Situation zu befreien. Die Gemeinschaft wiederum ist aufgerufen, diese Bemühungen zu unterstützen. Die Verletzung dieser Erwartungshaltung löst Irritationen aus, wie Erfahrungsberichte aus der Flüchtlingshilfe zeigen. Sabine Friedrich, die hier aktiv engagiert war, erzählt, dass sich viele der weiblichen Teilnehmerinnen in den Deutschkursen, die sie angeboten hat, nicht „bemüht“ hätten, weil sie „sowieso immer geglaubt haben, sie gehen eh nicht arbeiten“ (Int_12). Obwohl wir im Zusammenhang mit Geflüchteten problematische Assimilierungsanforderungen beobachtet haben – vor allem im Hinblick auf Arbeitsmoral, aber auch auf Werte wie die Gleichberechtigung von Männern und Frauen –, instrumentalisieren die Befragten dieses Typs die wahrgenommenen Irritationen nicht dazu, die Nichtaufnahme oder den Ausschluss von Geflüchteten zu fordern.

Die objektiven Klassenlagen der Befragten dieses Typs sind heterogener als bei den bisherigen Typen. Wir finden hier höhere Angestellte, Selbstständige und Beschäftigte im Dienstleistungsbereich, aber auch Personen aus nicht-privilegierten Familien, die einen sozialen Aufstieg gemeistert haben. Die Befragten dieses Typus weisen allerdings Ähnlichkeiten in ihren habituellen Dispositionen auf, wenn diese auch unterschiedlichen Laufbahnen entstammen. Dabei geht es insbesondere um die Erwartung, dass die einzelnen Gesellschaftsmitglieder Eigeninitiative und Anstrengungen zeigen. Während diese Orientierungen bei dem einen Teil der Befragten auf Erfahrungen des sozialen Aufstiegs zurückzuführen sind, der nur um den Preis großer Anstrengungen und Verzichte zu erlangen war, ist es bei den anderen ein unternehmerisches Selbstverständnis, das eng mit der eigenen beruflichen Position verbunden ist. Die Anforderung an Bedürftige, sich anzustrengen, um ihre Situation zu verbessern, ist in den moralischen Vorstellungen sowohl der Aufsteiger*innen als auch der Unternehmer*innen enthalten. Im Gegensatz zu Typ 2, der eher ein urbanes Milieu repräsentiert, leben die Befragten dieses Typs in ländlichen Regionen und wohnen teilweise nach wie vor in dem Dorf, in dem sie aufgewachsen sind. Auch das kann einen Einfluss darauf haben, wie die wechselseitigen Verpflichtungen von Individuum und Gemeinschaft wahrgenommen werden.

### Beitragsorientierte Solidarität (Typ 4)

Dieser Solidaritätstypus ist um die Moralität des Selbstverständnisses „wir, die hart Arbeitenden“ gebaut, die durch ihre Erwerbstätigkeit einen wichtigen Beitrag für die Gesellschaft leisten. Grundlage dieser Solidaritätskonfiguration ist das Leistungsprinzip, welches eng an die positive Konnotation von Erwerbstätigkeit gebunden ist. Der Bezug von sozialstaatlichen Leistungen wie Arbeitslosengeld oder Sozialhilfe werden hingegen negativ gesehen und nur in Ausnahmefällen als legitim erachtet. Die sozialstaatliche Solidargemeinschaft wird also als reziproke Leistungsgemeinschaft gefasst, in der diejenigen, die Bedarf an Transferleistungen haben, schnell unter Missbrauchsverdacht geraten können.

Die Einhaltung des Leistungsprinzips verlangt nicht nur eine angemessene Gegenleistung, sondern auch, dass es einen Unterschied zwischen jenen geben muss, die einen Beitrag leisten, und jenen, von welchen angenommen wird, dass sie das nicht in gleichem Ausmaß tun bzw. getan haben. Die Befragte Lina Wagner fasst diese Position zusammen mit: „Ich kann nicht für’s Nichtstun mehr bekommen als für’s Arbeitengehen.“ (Int_29) Das Arbeitslosengeld wird in dieser Lesart zu einem „Geschenk“ für Untätige, das die Anstrengungen der Erwerbstätigen entwertet und Ungerechtigkeitsgefühle hervorruft. Die Kritik an der Verweigerung entsprechender Leistungen für die Gemeinschaft zielt dabei zwar auch auf das gesellschaftliche „Oben“, was in den Gesprächen allerdings nur rudimentär präsent ist. So kritisiert Jan Wieninger, die „Reichen werden immer reicher“ (Int_31), und auch Michael Fuchs betont: „Die Geld haben, die kriegen dann wieder mehr. Reichensteuer gibt’s eh keine wirklich. […] Ja, das stimmt alles nicht, da passt das ganze System eigentlich nicht.“ (Int_13)

Allerdings stoßen wir bei Befragten dieses Typs in jenen Fällen auf Verständnis für den Bezug von Arbeitslosengeld, in denen ein zentrales Element des Leistungsprinzips, nämlich existenzsichernde Löhne, verletzt wird. Beispielhaft werden in den Gesprächen dafür wiederholt Frisör*innen genannt, eine Berufsgruppe mit traditionell niedrigen Einkommen. „Es kann nicht sein“, so Lina Wagner, „dass zum Beispiel eine Friseurin 1100 Euro oder so kriegt für 40 Stunden. Die arbeitet 40 Stunden und kann sich in Wahrheit keine 50-Quadratmeter-Wohnung leisten, den ganz normalen Standard, eine Wohnung, ein Auto, ein Handy, und ein bisschen ein Leben.“ (Int_29) Arbeitslosigkeit wird hier zur widerständigen Praktik gegen Ausbeutung, wie auch ein von Gabriel Drechsler berichteter Beispielfall zeigt. Er schildert die Geschichte eines Kochs, der netto nur 1200 Euro im Monat verdienen würde, „dafür, dass man 50 Stunden die Woche steht und sich abrackert. Ich verstehe das vollkommen, dass das unattraktiv ist, [...] wenn man für einen Hunderter weniger daheimsitzen kann und vielleicht im Pfusch [durch Schwarzarbeit, d. A.] irgendwas tun kann, nicht?“ (Int_08)

Der Fokus auf dem Leistungsprinzip führt bei diesen Befragten dazu, dass sich ihre Unterstützungsbereitschaft im Rahmen der nationalstaatlich organisierten Solidargemeinschaft auch auf Zugewanderte erstreckt. Allerdings wird dafür eine Unterwerfung unter die von ihnen vertretenen Leistungsnormen zur Voraussetzung gemacht. Das bedeutet in erster Linie, sich durch Erwerbstätigkeit selbstständig versorgen zu können und nicht von sozialstaatlichen Leistungen abhängig zu sein, wie Anna Nowak im Zusammenhang mit Geflüchteten deutlich macht: „Die, die ich wieder voll super finde, die herkommen, sagen: ‚Passt, ich bin jetzt da. Ich mache einen Deutschkurs. Ich gehe zum AMS, will eine Arbeit und fange zu arbeiten an.‘ Die sind für mich wirklich top. Also die können gerne dableiben.“ (Int_22) In dieser Logik argumentiert auch Lukas Aichinger, der es ablehnt, dass Asylwerber*innen, die eine Lehre machen, abgeschoben werden, denn „die eine Lehre machen und die Berufsschule und das alles schaffen, und dann kriegen sie auf einmal einen negativen Asylbescheid, das finde ich eigentlich blöd“ (Int_01). Gleichwohl bleibt die meritokratische Leistungslogik als Inklusionsprinzip im Kern prekär: zum einen, weil die Hegemonie des Erwerbsarbeitsethos andere Formen der Leistungserbringung ausklammert, zum anderen, weil sie auch die normativen Grundlagen für Ausgrenzung liefert, indem sie eine Einteilung in „Würdige“ – die Leistungserbringer und Leistungsbereiten – und „Unwürdige“ trifft (van Oorschot 2000). Fehlende (angenommene) Leistungsbereitschaft wird als Verletzung des Leistungsprinzips erfahren und mit starken Ausgrenzungsforderungen beantwortet. „Für mich sind das die Schlimmen“, so expliziert dies Anna Nowak, „die einfach herkommen, nichts arbeiten wollen, sich nicht integrieren, gar nichts. [...] Weil für was sind die da? Für gar nichts. Dass sie unsere Steuergelder nehmen, und weiß ich nicht, jeden Monat ein neues Handy oder sonst irgendwas kaufen. Und wir rackern uns ab, bis wir umfallen.“ (Int_22). Die nur „herumsitzen, die nur lästig sind“, so Lukas Aichinger, sollten wieder in ihre Herkunftsländer müssen (Int_01).

Die Befragten dieses Typus teilen eine einigermaßen homogene sozialräumliche Lage. Der überwiegende Teil entstammt einem ländlichen handwerklichen bzw. bäuerlichen Milieu. Sie verfügen über solide Berufsausbildungen entweder in Form von Lehrabschlüssen oder berufsbildenden weiterführenden Schulen. Sie arbeiten in typischen Handwerks- und Arbeiterberufen, einige hat die Ausbildung auch in eine Angestelltenlaufbahn geführt. Die Gruppe teilt nicht nur die Erfahrung einer ähnlichen sozialen Lage, sondern auch des Lebensabschnitts, denn sie gehören alle – mit einer Ausnahme – einer jüngeren Generation im Alter von 20 bis 32 Jahren an. In der Betonung des hohen Stellenwerts von Erwerbsarbeit und damit verbundener Werte wie harte Arbeit, Ehrlichkeit, Autonomie etc. referenziert diese Gruppe auf Moralvorstellungen von Fraktionen der traditionellen Arbeiter*innen-Klasse, wie sie u.a. von Sennet und Cobb (1993), Michèle Lamont (2000) oder Vester et al. (2001) beschrieben wurden. Diese Moralität der harten Arbeit ist nicht nur der basalen existenzsichernden Bedeutung von Erwerbsarbeit in dieser Klassenlage geschuldet: Sie stellt darüber hinaus einen zentralen Eckpfeiler der Anerkennung dar. Diese habituellen Dispositionen schaffen nicht nur die Grundlage für Zugehörigkeit und Identitätsbildung, sie legitimieren auch den Ausschluss von anderen („Autochthone“ und Zugewanderte gleichermaßen), die diesen normativen Standards scheinbar nicht genügen.

Als Angehörige eines ländlichen, handwerklichen Milieus sind die Befragten von kollektiven Abstiegserfahrungen betroffen. Das betrifft zum einen die ökonomische Lage: Die Löhne von Handwerker*innen und Arbeiter*innen stagnieren seit den 1990er-Jahren oder nehmen sogar ab (Rechnungshof 2016). Zum anderen sind ihre Bildungsabschlüsse angesichts einer Tendenz zu höherer Bildung von Entwertung bedroht. Diese Realität wird auch von Erfolgsgeschichten auf der individuellen Ebene nur bedingt ausgeglichen. So ist beispielsweise das Grundmotiv im Gespräch mit Lukas Aichinger die fehlende Anerkennung seiner Arbeit durch die Gesellschaft und die mangelnde Vertretung, obwohl er heute ein erfolgreicher Landwirt ist. Diese Erfahrungen von materieller und symbolischer Entwertung verletzen ein auf dem Leistungsprinzip basierendes Gerechtigkeitsgefühl. Entsteht dann der Eindruck, dass es sich andere leichter machen, wird das als Provokation empfunden und mit Ausgrenzung reagiert.

### Moralisierend autoritäre Solidarität (Typ 5)

Die Befragten dieses Typs betrachten die Gesellschaft als moralische Ordnung. Während die beitragsorientierte Gruppe die Unterwerfung unter das Leistungsprinzip als zentrale Bedingung für die Zugehörigkeit zur Solidargemeinschaft sieht, fordern die von uns als „moralisierend autoritär“ typologisierten Befragten darüber hinaus die Unterwerfung unter eine moralische Ordnung. Grundsätzlich wird von diesen Befragten zwar Menschen, die unverschuldet ihren Arbeitsplatz verlieren, sozialstaatliche Unterstützung zugestanden, diese Fälle werden jedoch als Ausnahmen wahrgenommen. Mehrheitlich wird Erwerbslosigkeit als Folge von Unwilligkeit beschrieben, die in einer Charakterschwäche der Person begründet liege. „Hat jemand eine schwere Krankheit und kann nicht mehr arbeiten“, so Erwin Staudinger, „das ist dann okay. Aber einfach nur aus Faulheit sozusagen, dass einer jahrelang Geld kriegt für’s Nichtstun, das finde ich nicht gut.“ (Int_27) Josef Alp ist davon überzeugt: „Wer arbeiten will, kriegt eine Arbeit“ (Int_02). Andernfalls seien die Leute eben zu „faul“ (so auch Int_15, Int_04, Int_27) oder zu „stolz“ (Int_28). Diese Pflicht zur Arbeit wird auch von Zugewanderten verlangt, denn auch ohne Deutsch-Kenntnisse könne man etwas beitragen, wie Sandra Vordermeier meint: „Straßen kehren, das kann eigentlich jeder, der körperlich halbwegs gut beisammen ist.“ (ebd.)

Die Befragten dieses Typus sehen Erwerbsarbeit weniger unter dem Gesichtspunkt der Leistung und des Beitrags, sondern verbinden sie vielmehr mit Anständigkeit und Konformität. Entsprechend wird Erwerbslosigkeit als Verstoß gegen geltende Normen gewertet und darf keine Solidarität erwarten. Die sozialstaatliche Arbeitslosenunterstützung wird deshalb als Leistung betrachtet, die nur für „Härtefälle“ gewährt werden soll, wie das folgende Zitat von Petra Beer zeigt: „Ich meine, du kannst keinen verhungern lassen, [...] aber warum gibt es Unterstützung für die, die einfach zu faul sind, dass sie arbeiten gehen, zu bequem sind?“ (Int_04) Hier zeigt sich eine stark eingeschränkte Reichweite der Solidarität, die eine große Zahl an Erwerbsarbeitslosen ausschließt. Durch Sanktionen und Kürzungen solle dafür Sorge getragen werden, dass Erwerbslose wieder in Beschäftigung gelangen. Autoritäre Haltungen treten bei diesem Thema deutlich hervor. Solange sich Arbeitslose „Luxus“ wie Alkohol oder Zigaretten leisten könnten, sei das Arbeitslosengeld zu hoch (ebd.). Zur Aufrechterhaltung der normativen Ordnung wird der Staat in der Pflicht gesehen; so fordert Gerald Hofer beispielsweise „härtere Regulative“ (Int_15). Und auch Josef Alp betont, „härter durchgreifen“ müsse man dort, wo jemand „von sich aus nicht arbeiten will“, „wenn einer jetzt z.B. Jobs einfach von sich aus ablehnt, weil er nicht mag. Arbeiten muss man für die Gemeinschaft.“ (Int_02)

Die Solidargemeinschaft wird hier als eine Gemeinschaft geteilter Werte und Normen gesehen, auf deren Einhaltung geachtet werden muss, die allerdings als von unterschiedlichen Seiten bedroht wahrgenommen wird. Neben einer mangelhaften Arbeitsmoral wird auch Zuwanderung als Ursache ausgemacht. So zeigt sich Gerald Hofer besorgt, „was die kulturelle und vor allem religiöse Walze oder Welle betrifft“ (Int_15). Zwar gibt es eine gewisse Offenheit hinsichtlich Zuwanderung, wie ein weiteres Zitat von Gerald Hofer zeigt, diese dürfe aber nicht die eigenen Werthaltungen gefährden: „Und ich glaube halt einfach, dass Mobilität, offene Grenzen, das ist ganz normal, dass Kulturen verschwimmen, ja, aber im Prinzip sollen schon die Werte oder die Grundwerte von dem Land, wo man hingeht, erhalten bleiben, ja?“ (ebd.) Er hat den Eindruck, dass die Politik sich in Österreich in den letzten Jahren zu wenig „auf die Füße gestellt“ habe.

Was die objektive Klassenlage betrifft, sind die Personen dieser Gruppe in Angestelltenpositionen erwerbstätig, haben Matura oder höhere Bildungsabschlüsse und stammen aus ländlichen oder kleinstädtischen Umgebungen. Mit ihrem Ethos der Pflichterfüllung und ihren zentralen Werten der Disziplin, Ordnung und Verlässlichkeit erinnern sie an die Beschreibungen eines kleinbürgerlichen Arbeitnehmer*innen-Milieus bei Michael Vester et al. (2001). Die Radikalisierung der Werthaltungen hin zu autoritärer Ausgrenzung in diesem kleinbürgerlichen Milieu interpretieren Norris und Inglehart (2018) als Gegenreaktion auf die kulturellen Veränderungen in Anschluss an 1968. Die früher dominante, kulturelle Mehrheit wurde demnach schrittweise zur Minderheit, vormals hegemoniale Normen und Werte sind seither in der Gesellschaft nicht mehr umfassend respektiert (ebd. S. 87). Ähnlich argumentiert Reckwitz (2017), dass die traditionelle Mittelschicht am Dominanzverlust ihres Lebensstils, ihrer Werte und Normen als Repräsentationen einer gesellschaftlichen Normalität zu leiden scheint. Dem entspricht, dass die Befragten dieses Typs sehr stark aus der Defensive argumentieren und auf den Erhalt einer früheren, in die Krise geratenen moralischen Ordnung fixiert sind.

### National ausgrenzende Solidarität (Typ 6)

Der von uns unter dem Begriff der „national ausgrenzenden Solidaritätsorientierung“ gefasste Typ 6 lässt sich unter einer klassenanalytischen Brille als Variante des weiter oben beschriebenen Typs 4 betrachten, weshalb hier die Darstellung im Beitrag etwas kürzer ausfällt. Die hier zusammengefassten Befragten weisen eine ähnliche objektive Klassenlage auf.^9^ Sie unterstützen einen starken Sozialstaat, haben aber die Erfahrung gemacht, dass der Staat sich nicht ausreichend um diejenigen kümmert, „die es schwer haben“. Zugleich haben sie die angeblich begrenzte Finanzierbarkeit des Sozialstaates als Erklärung dafür akzeptiert. Vor diesem Hintergrund wirkten die medial aufgebauschten Hilfeleistungen an Geflüchtete in den Jahren 2015 und 2016 auf sie als Provokation: „Ich meine, ohne dass das jetzt rassistisch oder irgendwas klingt, aber ich bin halt eigentlich eher dafür, dass es in Österreich auch genug Leute gibt, denen geholfen gehört, und dass man sich halt eigentlich um die eigenen Leute vielleicht ein wenig mehr umschauen sollte.“ (Int_14)

Das Muster der „national exkludierenden Solidarität“ basiert dabei auf der Wahrnehmung, dass geflüchtete Menschen mehr Aufmerksamkeit, finanzielle Zuwendung und Unterstützung bekommen als Menschen in Österreich, die ebenfalls Hilfe brauchen. Diese Wahrnehmung einer Umverteilung hin zu den Zugewanderten löst Ungerechtigkeitsgefühle aus. Aufbauend auf einer klaren Trennung in „die Einheimischen“ und „die Anderen“ wird gefordert, dass sozialstaatliche Leistungen primär den Einheimischen zugutekommen sollten. An die Stelle des Leistungsprinzips der „beitragsorientierten Solidarität“ von Typ 4 tritt hier eine Art „Wohlfahrtschauvinismus“. Zwar solle man Menschen, die vom Krieg betroffen sind, helfen, aber Österreicher*innen hätten Anspruch auf mehr: „Ich meine, es kann nicht sein, dass denen mehr bleibt als unsereins.“ (Int_11) Eine ähnliche Klassenlage – die Verortung „unten“ in der Gesellschaft und die Erfahrung kollektiven Abstiegs – kann also zwei recht unterschiedliche Solidaritätskonfigurationen stützen. Der Unterschied liegt primär darin, ob die Betonung des Leistungsprinzips die Ausgrenzung entlang von Staatsbürgerschaft und nationaler Zugehörigkeit aushebelt oder nicht.

### Ethno-nationale Ausgrenzung (Typ 7)

Ähnlichkeiten wie zwischen Typ 4 und 6 lassen sich hinsichtlich der Klassenperspektive auch zwischen dem eben beschriebenen Typ 5 und dem Typ 7 ausmachen. Bei Letzterem haben wir es jedoch mit einer deutlichen Radikalisierung der Exklusion und damit verbundener Deutungen im Sinne eines kulturalisierenden Rassismus zu tun. So wurde in den hier betrachteten Interviews viel über „Kopftuchträgerinnen“ geklagt und darüber, dass man „kein Deutsch mehr höre“. Auch diese Befragten drücken Verständnis dafür aus, dass Kriegsflüchtlingen geholfen werde. Aber „sobald das vorbei ist, wieder zurück in die Heimat. Die haben hier keine Wurzeln.“ (Int_10)

In diesem Typus finden wir Personen mit guter – teils akademischer – Bildung, die den Sozialstaat für die von ihnen behauptete Umverteilung von den „Inländern“ zu den „Ausländern“ kritisieren. Es finde, so ihre Vorstellung, eine Verschiebung von beitragsbasierten zu bedarfsorientierten sozialstaatlichen Leistungen statt, die angeblich Migrant*innen nützt und zum Schaden der eigenen Statusgruppe ausfällt. Während die Befragten des Typs „moralisierend autoritäre Solidarität“ vor allem für die Durchsetzung eines traditionellen Arbeitsethos eintreten und vor diesem Hintergrund Skepsis gegenüber Zuwanderung äußern, finden sich in der hier betrachteten Gruppe offen kulturrassistische Haltungen, auch unabhängig vom Aspekt der Leistungsbereitschaft. „Unter uns bleiben“ ist das Motto in Bezug auf jene, die ihre „Wurzeln“ im Land haben. Zugleich sind sozialdarwinistische Orientierungen erkennbar: Man will den Sozialstaat für die Mitglieder des nationalen Kollektivs reservieren, die es geschafft haben und sich selbst als „Leistungsträger“ bezeichnen. „Für Ausländer“, so der Befragte Gerhard Meier in diesem Sinne, „sollte es keine Leistungen geben, dann ersparen wir uns was, und die Österreicher könnten mehr bekommen. Aber besonders die, die eben viel leisten, viel Bildung haben oder schon lange gearbeitet haben.“ (Int_19)

Die extreme Einengung der Reichweite der Solidarität auf eine Kampfsolidarität der Bessergestellten wird gestützt durch die Abwertung von Migrant*innen insbesondere türkischer Herkunft, denen Leistungsfähigkeit und Leistungsbereitschaft generell abgesprochen wird, wie die Aussagen Konrad Schweighofers zeigen: „Bei den anerkannten Flüchtlingen wird das so sein wie bei den Türken, da finden 95 Prozent keinen Job. Die werden immer von der Sozialhilfe oder der Mindestsicherung leben.“ (Int_26) Er verwendet dafür den Begriff der „einseitigen Solidarität“ im Sinne von „wenn ich immer nur nimm, nimm, nimm und nie die Gelegenheit habe, was zurückzuzahlen“.

## Diskussion der Ergebnisse und Schlussfolgerungen

In diesem Beitrag haben wir auf der Grundlage von Daten aus einer umfassenden empirischen Untersuchung analysiert, wie sich Solidaritätsorientierungen hinsichtlich des Sozialstaats und der Unterstützung von Flüchtlingen in der österreichischen Gesellschaft unterscheiden. Ein besonderer Fokus lag dabei auf der Klassenlage der Befragten bzw. deren Manifestation in den Interviewäußerungen. Aufbauend auf der Bourdieu’schen Konzeption sozialer Klassen und ihrer Weiterentwicklung im Konzept des „boundary making“ durch Lamont und andere haben wir neben der „objektiven“ sozialen Lage vor allem den Klassenhabitus mit seinen latenten Zugehörigkeiten, Identifikationen und Distinktionen in den Blick genommen. Wie dargestellt, verstehen wir unter Solidarität eine Form der Bindung zwischen Menschen, die sich in einer (Selbst‑)Verpflichtung zum gegenseitigen Beistand manifestiert – ob aktiv in Form eigeninitiativer Unterstützung oder latent in Form der Akzeptanz wohlfahrtsstaatlicher Transferpolitiken. Im Zentrum stand die im Sozialstaat institutionalisierte Form der Solidarität.

Es konnte gezeigt werden, dass es zwischen der Klassenlage und den Solidaritätsorientierungen insbesondere im Hinblick auf den Sozialstaat klare Zusammenhänge gibt. Wir können also den Schluss ziehen, dass die soziale Klassenlage einen Einfluss auf Solidaritätskonfigurationen hat. Dies ist am Beispiel des Typus der „politischen Solidarität“ am deutlichsten zu erkennen: Hier geht mit einer prekären objektiven Lebenslage ein ausgeprägtes und reflektiertes Klassenbewusstsein einher, das sich, wie gezeigt, in einer universalistischen Solidaritätsorientierung niederschlägt. Die wahrgenommenen Gemeinsamkeiten aller Benachteiligten über nationalstaatliche Grenzen hinweg sind Wertgrundlage eines gemeinsamen Engagements für mehr und letztlich weltgesellschaftliche Gerechtigkeit. Unter umgekehrten Vorzeichen zeigt sich auch im Falle der unter „altruistische Solidarität“ gefassten Befragten die Bedeutung der Klassenlage für die Solidaritätsorientierung: Die sehr privilegierte Lebenslage in urbanen Milieus geht mit einem großen Abstand zu jenen im Land einher, die auf Umverteilung und Unterstützung angewiesen sind. Die Befragten dieses Typs nehmen an, dass Letztere vom Sozialstaat, den sie durchaus befürworten, ausreichend versorgt werden. Aufgrund der sozialen Distanz haben sie aber keinen Einblick in deren Lebenswelten. Ihre ebenso universalistische, jedoch stärker karitativ als politisch grundierte solidarische Haltung gegenüber Geflüchteten klingt hingegen eher nach „noblesse oblige“ und verweist auf einen kosmopolitisch-philanthropischen Habitus.

Im Typus der „beitragsorientierten Solidarität“ finden wir Fragmente einer ländlichen, jüngeren Arbeiter*innen- und Handwerksklasse, die zwar an den negativen Ausformungen des Kapitalismus leidet, diese Kränkungen aber nur rudimentär als Klassenerfahrungen einordnet. Leistungsbasierte Ausgrenzungsmechanismen sind bei ihnen mit ethnischen Grenzziehungen in einem komplexen Verhältnis verbunden. Während die Orientierung am Leistungsprinzip und eine grundsätzliche Solidarität unter den produktiv Beschäftigten eine aktiv nach ethnisch-nationalen Kriterien erfolgende Ausgrenzung in dieser Gruppe unterbindet, bleiben derartige Grenzziehungen über das von den Massenmedien vermittelte Bild der großzügig versorgten, aber wenig arbeitsbereiten Migrant*innen dennoch latent (vgl. dazu auch Dörre et al. 2013). Beim Typ der „ermöglichenden leistungsorientierten Solidarität“ lassen sich die habituellen Dispositionen auf unterschiedliche Laufbahnen zurückführen: Dass man von allen Anstrengung und Eigeninitiative fordert, fußt bei den einen auf Erfahrungen des sozialen Aufstiegs, bei den anderen auf einem unternehmerischen Selbstverständnis. In den Aussagen der Vertreter*innen einer ruralen und kleinstädtischen kleinbürgerlichen Dienstleistungsklasse einer „moralisierend autoritären Solidarität“ wiederum spielen ein Ethos der Pflichterfüllung und Werte der Disziplin, Ordnung und Verlässlichkeit eine zentrale Rolle.

In allen drei Gruppen der Typen 3, 4 und 5 lassen sich somit eine deutliche Selbstidentifikation mit der „erwerbstätigen Bevölkerung“ und ein damit assoziierter Leistungsethos beobachten, der die Grundlage bildet für eine Solidaritätsorientierung, in deren Mittelpunkt eine symmetrische Reziprozitätserwartung an Bedürftige steht. Ethnisch-nationale Grenzziehungen bleiben eher implizit; prägend für die eigene Solidaritätsorientierung sind die Bereitschaft der nehmenden Seite zur Selbsthilfe sowie die damit assoziierte Selbstverpflichtung, sozialstaatliche Infrastrukturen nicht zu missbrauchen (siehe auch Beck und Westheuser in diesem Heft). Im Falle der unter Typ 6 und 7 versammelten Zeugnisse haben sich diese Prioritäten verkehrt: Hier wird die arbeitende Bevölkerung explizit national bzw. ethnisch-national indexiert und eine Grenze gezogen zwischen Bedürftigen, die die „eigene“ Herkunft teilen, und solchen, die den österreichischen Sozialstaat illegitimerweise als „Fremde“ in Anspruch nehmen. Ausschlaggebend für die Solidaritätsorientierung ist vordringlich die Zugehörigkeit der nehmenden Seite zum nationalen bzw. ethnischen „Wir“; Leistungsbereitschaft und moralische Integrität spielen zwar ebenso eine wichtige Rolle, können aber eine „falsche“ Herkunft nicht kompensieren.

Summarisch zeigt sich damit ein klarer Zusammenhang zwischen Klassenlage einerseits und Solidaritätsorientierung andererseits. So unterscheiden sich die Typen 1 und 2 zwar hinsichtlich ihrer ökonomischen Lage manifest, jedoch teilen beide eine „universalistische“ Orientierung, wenn auch unter stark divergierenden Vorzeichen hinsichtlich einer eher politisch-egalitären und einer eher karitativ-paternalistischen Motivation. Bei den Angehörigen der mittleren Volksklassen der Typen 3–6 steht die Reziprozitätserwartung im Mittelpunkt, die indes hinsichtlich der Dimensionen Leistung, Moral und Herkunft deutlich variiert. Ein klarer Zusammenhang ist allerdings nicht gleichbedeutend mit einer *determinierenden* Kausalität: Geteilte objektive Klassenlagen führen nicht notwendigerweise zu denselben Orientierungen (Sayer 2005, S. 51). So stellten wir beim Typ der „beitragsorientierten Solidarität“ und jenem der „national exkludierenden Solidarität“ ähnliche objektive Klassenlagen fest, insofern die Befragten aus der Arbeiter*innenklasse und handwerklichen Milieus stammten und einen kollektiven Abstieg erlebt hatten. Die Solidaritätskonfigurationen dieser beiden Typen unterscheiden sich jedoch recht deutlich: Je nachdem, ob dem Leistungsprinzip oder dem nationalstaatlichen Statusprinzip Priorität eingeräumt wird, ist die Reichweite der Solidarität sehr unterschiedlich. Umgekehrt sind bisweilen auch ähnliche Solidaritätskonfigurationen bei Personen unterschiedlicher Klassenlagen vorzufinden. So erwiesen sich die sozialen Positionen des Typs der „ermöglichenden leistungsorientierten Solidarität“ als heterogener als die der anderen Typen. Es besteht also sowohl einerseits ein deutlicher Zusammenhang zwischen sozialer Klasselage und Solidaritätsorientierung als auch andererseits eine relative Offenheit in dieser Hinsicht.

Dies verweist auf Ambivalenzen in den Solidaritätsvorstellungen. Solche Ambivalenzen sind insofern naheliegend, als die soziale Herkunft, die Erfahrungen im Lebensverlauf und die aktuelle Lebenssituation zusammengenommen durchaus zu widersprüchlichen Dispositionen führen können. Darüber hinaus können ähnliche Dispositionen situativ unter dem Einfluss von öffentlichen Diskursen unterschiedliche Solidaritätsorientierungen stützen. Insbesondere die „beitragsorientierte Solidarität“ erwies sich als weitgehend ambivalent, insofern leistungsbasierte Inklusions- und Ausgrenzungsmechanismen mit ethnischen Grenzziehungen in einem komplexen Verhältnis verbunden sind.

Die vergleichende Auswertung von Solidaritätsorientierung und sozialer Position verdeutlicht damit die anhaltende Bedeutung einer klassensensiblen Perspektive, wie sie insbesondere in der deutschsprachigen Soziologie in den letzten Jahrzehnten vernachlässigt wurde, wodurch vertikale Klassifikations- und Spaltungslinien tendenziell unsichtbar blieben und die Erfahrungen der dominierten Klassen aus dem Blick gerieten und entwertet wurden. Das stand schon in den 1990er-Jahren im Widerspruch zu einer empirischen Realität, die Benachteiligung und Ausgrenzung entlang der sozialen Herkunft produzierte. Obwohl jüngere populäre Erfahrungsberichte mit Klassismus (u.a. von Didier Eribon 2016) und bestimmte gesellschaftliche Ereignisse (z.B. die COVID-19-Krise) dem Klassenbegriff im öffentlichen und wissenschaftlichen Diskurs wieder mehr Auftrieb verliehen haben, bleibt er als analytische Kategorie im deutschsprachigen Kontext weiterhin marginalisiert. Diese „Klassenvergessenheit“ (Williams 2017; Dörre et al. 2018) hat über Deutungsweisen der gesellschaftlichen Wirklichkeit nicht nur einen Einfluss auf gesellschaftspolitische Auseinandersetzungen, sie schränkt auch sozialwissenschaftliche Erkenntnismöglichkeiten ein. Für die dominierten Klassenlagen bedeutet diese De-Thematisierung, dass die damit verbundenen Erfahrungen von Unterdrückung, Ausbeutung und Entwertung nicht angemessen artikuliert werden können.

Die jüngeren politischen Verschiebungen hin zu einer rechts-konservativen Hegemonie haben wiederum einer Ethnisierung der sozialen Frage Vorschub geleistet, die die „Einheimischen“ gegen „Ausländer*innen“ anstelle von „oben“ gegen „unten“ in Stellung bringt. Die hier dargestellten Forschungsergebnisse machen jedoch deutlich, wie wichtig es in diesem Kontext ist, die verschüttete Klassenperspektive zu revitalisieren: Sie zeigen, dass es für die Erklärung von Solidaritätskonfigurationen – und wohl auch anderer politischer Orientierungen – sinnvoll ist, auf die Kategorie der sozialen Klasse zurückzugreifen. Dabei hat sich, wie gesehen, ein Klassenkonzept als fruchtbar erwiesen, das neben der objektiven sozialen Lage auch den Klassenhabitus und das Klassenbewusstsein als analytische Dimensionen berücksichtigt. Diese Einsicht ist umso relevanter, als es in konkreten politischen Situationen und durch vorherrschende Rahmungen im öffentlichen Diskurs immer dort zu Verschiebungen in den Solidaritätsorientierungen kommt, wo wir es nicht mit festgefügten und teils extremen Haltungen zu tun haben. Und das betrifft die große Mitte zwischen den Polen des dargestellten Solidaritätskontinuums, welche grundsätzlich für unterschiedliche Haltungen zu gewinnen ist und wo verschiedene Klassenfraktionen mit gleichen Botschaften angesprochen werden können.

## Footnotes

1 In der Literatur wird allerdings darüber debattiert, ob auch *verrechtlichte *Unterstützungsleistungen unter den Solidaritätsbegriff fallen. Das gilt insbesondere für Autor*innen, die Solidarität als freiwillige, aktive Handlungsleistung begreifen; vgl. dazu u.a. Bierhoff und Küpper (1999).

2 Das Projekt wurde vom Österreichischen Wissenschaftsfonds (Nr. I2698-G27) und dem nationalen Forschungs‑, Entwicklungs- und Innovationsbüro in Ungarn (NKFIA, ANN_2016/1, 120360) gefördert.

3 Österreich zählt zu den konservativen Wohlfahrtsregimes, das sozialstaatliche Absicherung mit der Betonung der Statussicherung verbindet, wodurch Ungleichheiten aus der Erwerbssphäre reproduziert werden (Tálos und Obinger 2020). Dass die neoliberale Wende abgefedert und ein gravierender Sozialabbau vermieden wurden, ist durchaus auf die Sozialpartnerschaft (Tálos 2008) zurückzuführen, die die politische Realität in Österreich seit dem Zweiten Weltkrieg wesentlich geprägt hat – auch wenn die sozialpartnerschaftliche Konzertierung und insbesondere die Einbindung von Gewerkschaften und Arbeiterkammern in die staatliche Politik unter den konservativ-rechtspopulistischen Regierungen seit dem Jahr 2000 deutlich sistiert war.

4 Man kann die beiden Logiken noch um eine Logik des Androzentrismus ergänzen, da Frauen in der sozialstaatlichen Ordnung in zentralen Aspekten wesentlich benachteiligt waren und in erheblichem Maße noch immer sind.

5 Dieser Solidaritätstypus zeigt Ähnlichkeiten mit Formen von Solidarität, die in der Literatur als „Kampfsolidarität“ (Bayertz 1998, S. 41) oder „politische Solidarität“ (Scholz 2008) bezeichnet werden.

6 Vergleichbar mit der deutschen Bundesagentur für Arbeit.

7 Die Verbindung von Altruismus und Solidarität mag auf den ersten Blick widersprüchlich erscheinen. Wir rekurrieren hier unter anderem auf eine Bezeichnung von Eckhart Voland (1999), der neben gemeinsamen Interessen auch Altruismus, also die Verpflichtung, Benachteiligten zu helfen, als Grundlage für Solidarität benennt. Der Typus verweist darauf, dass in der Praxis die Grenzen zwischen Fürsorge und Solidarität oft fließend verlaufen. So ist einerseits die Nähe zu Wohltätigkeit nicht von der Hand zu weisen ist, andererseits beziehen die Befragten klar Position für sozialstaatliche Umverteilung. Darüber hinaus politisierten sich einige der Befragten im Laufe ihres Engagements in der Flüchtlingshilfe in einer Weise, die sie näher an den Typ 1 rücken lassen.

8 So das vom ehemaligen deutschen Bundesminister für Wirtschaft und Arbeit, Wolfgang Clement, geprägte Leitmotiv der Hartz-IV-Reformen; in den Interviews wird indes, anders als in der damaligen Debatte, das „Fördern“ stärker gewichtet als das „Fordern“.

9 Aufgrund der geringen Fallzahl ist die Typologie in diesem Fall (wie auch im Fall von Typ 7) empirisch weniger robust. Aufgrund der im selben Forschungsprojekt durchgeführten Repräsentativerhebung ist jedoch zu vermuten, dass es sich hierbei um ein durchaus verbreitetes Muster einer Solidaritätskonfiguration handelt.
